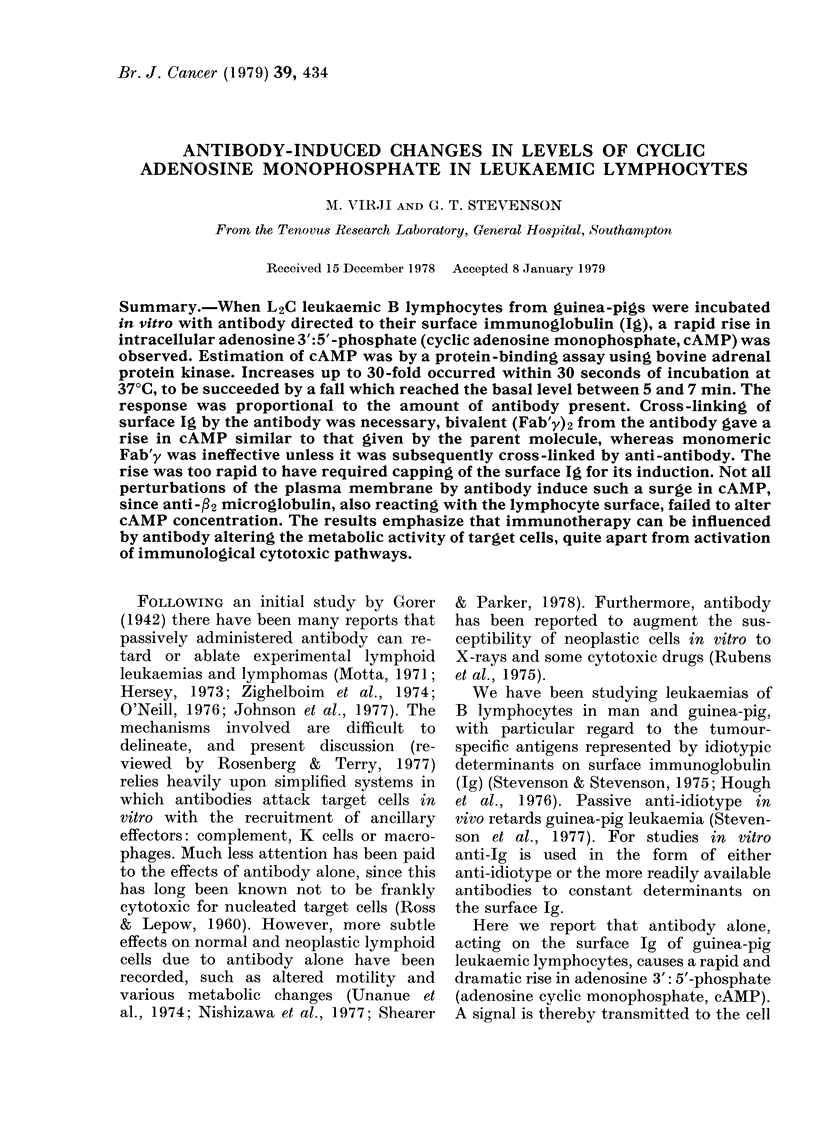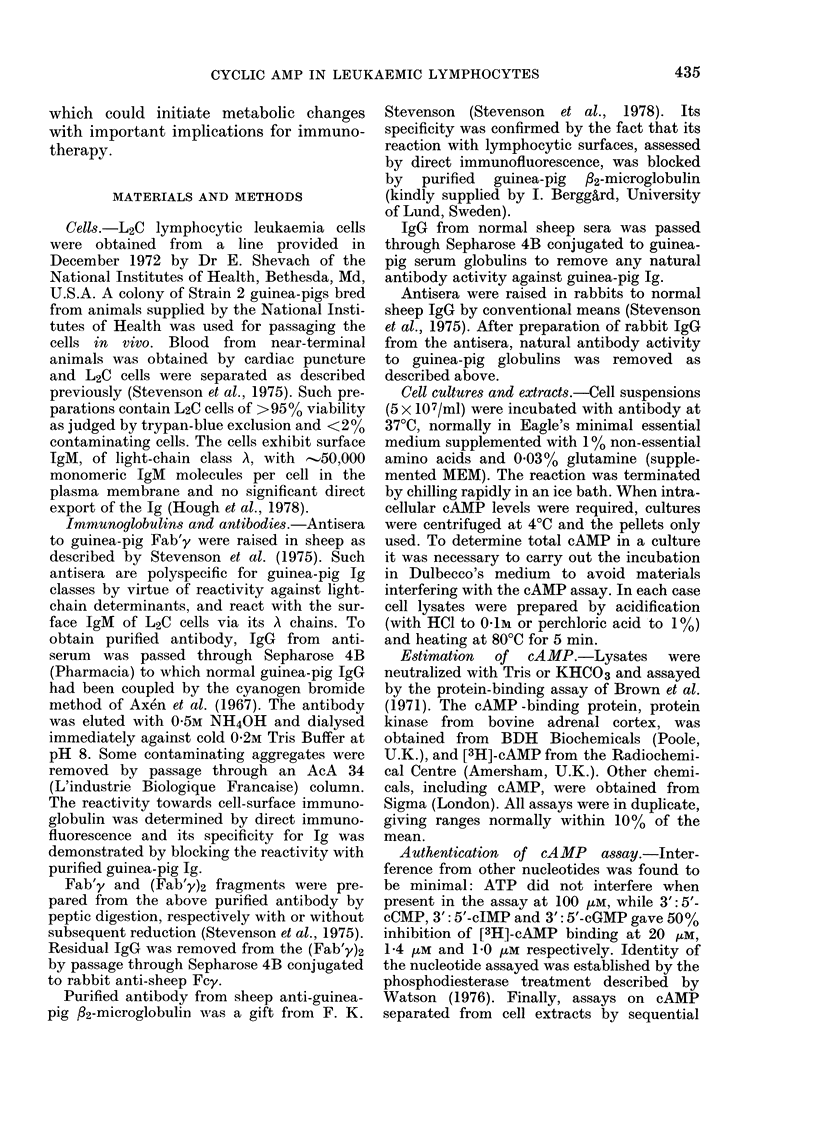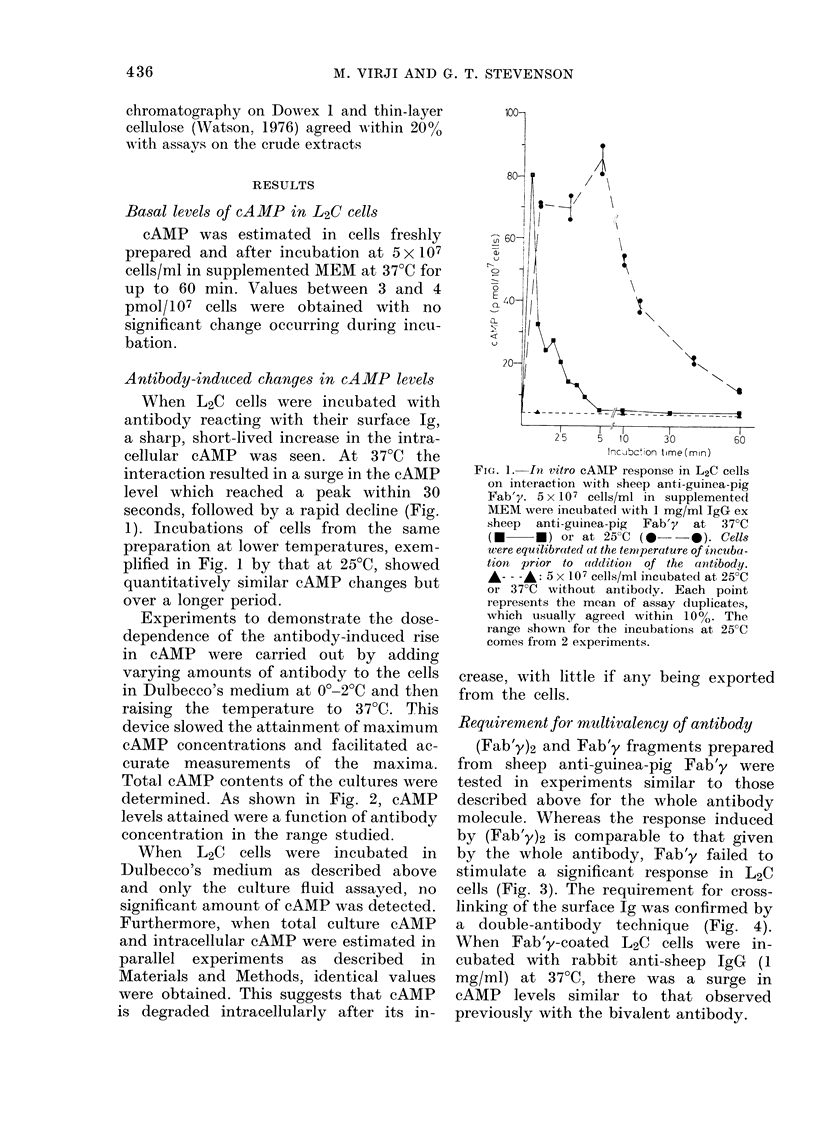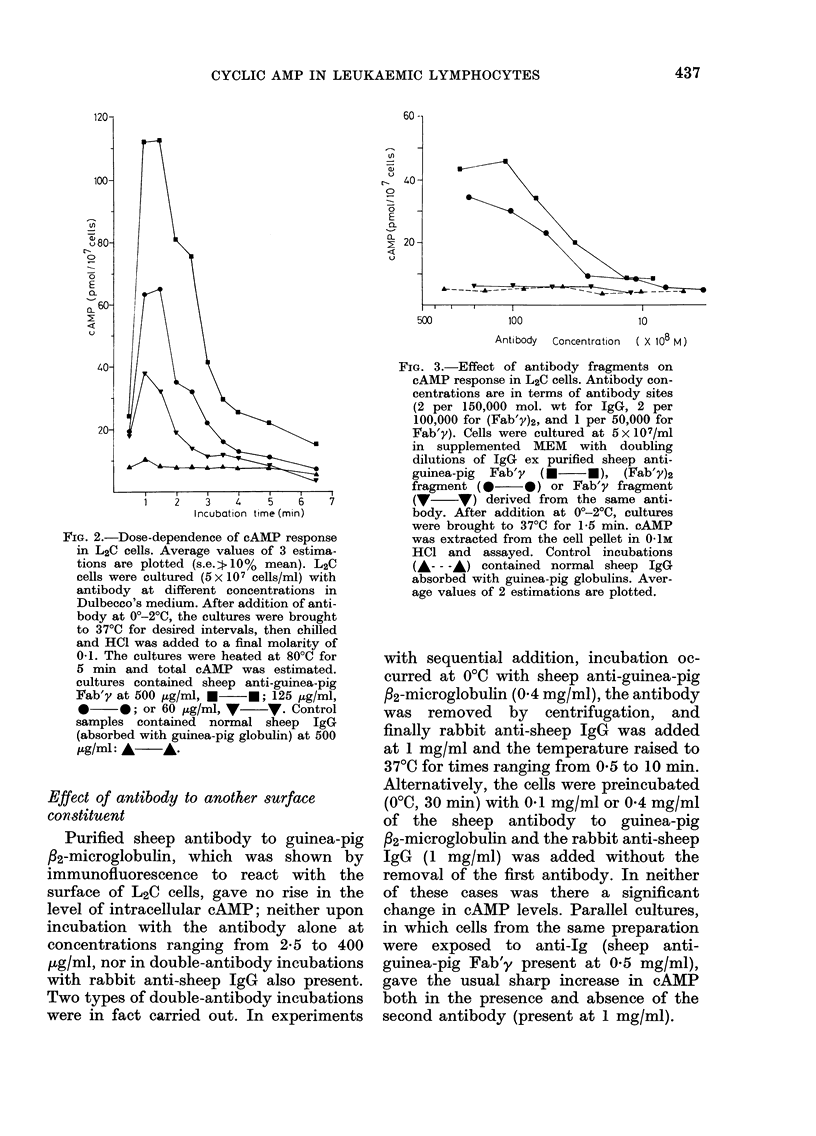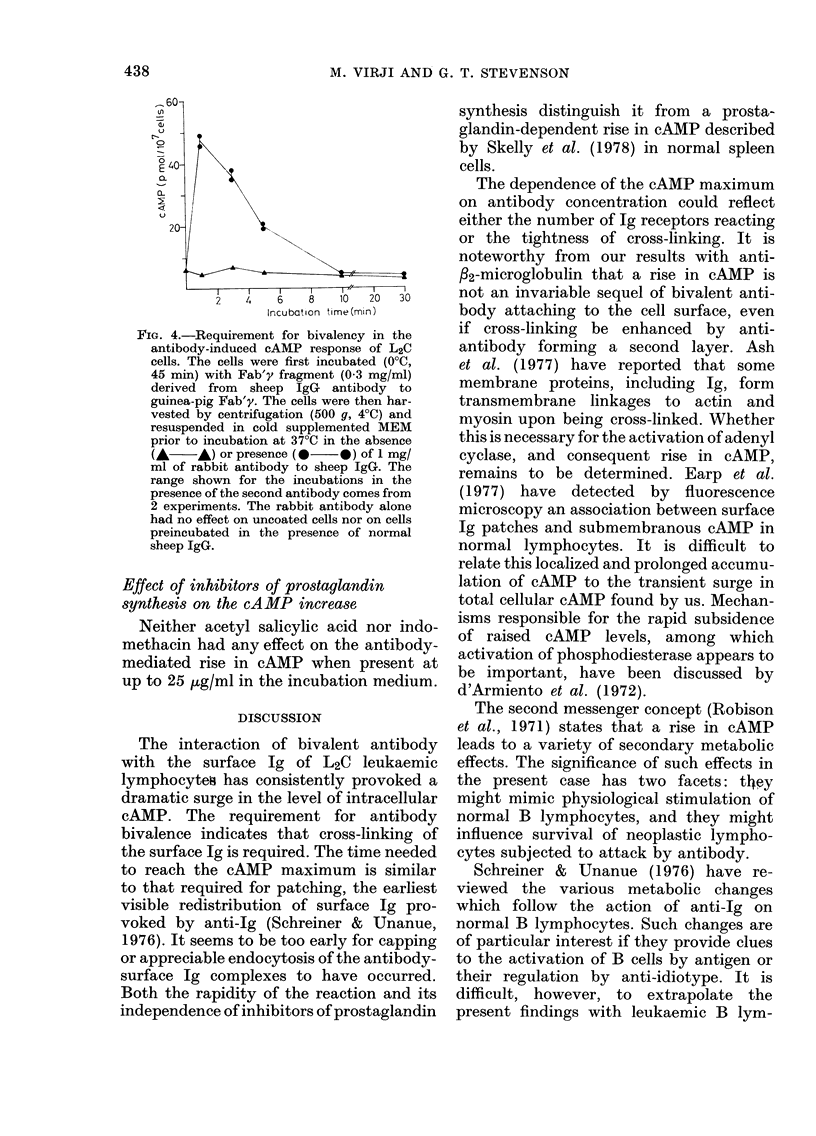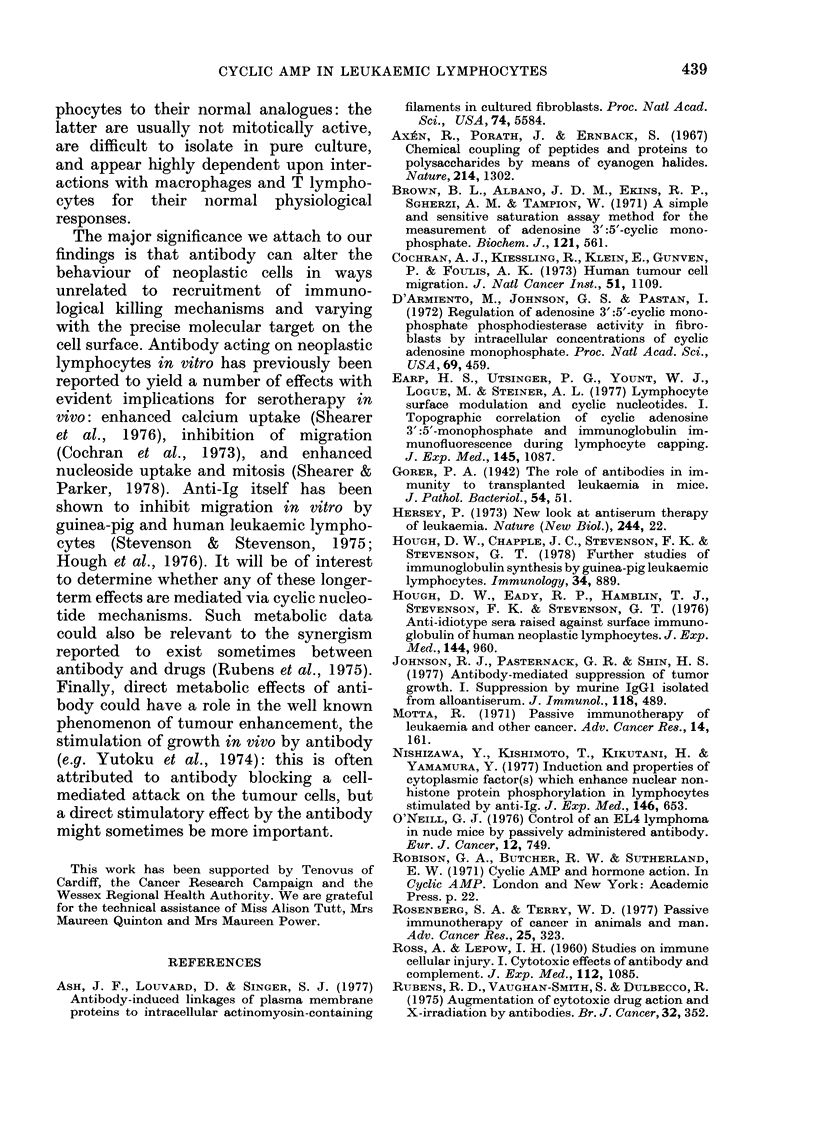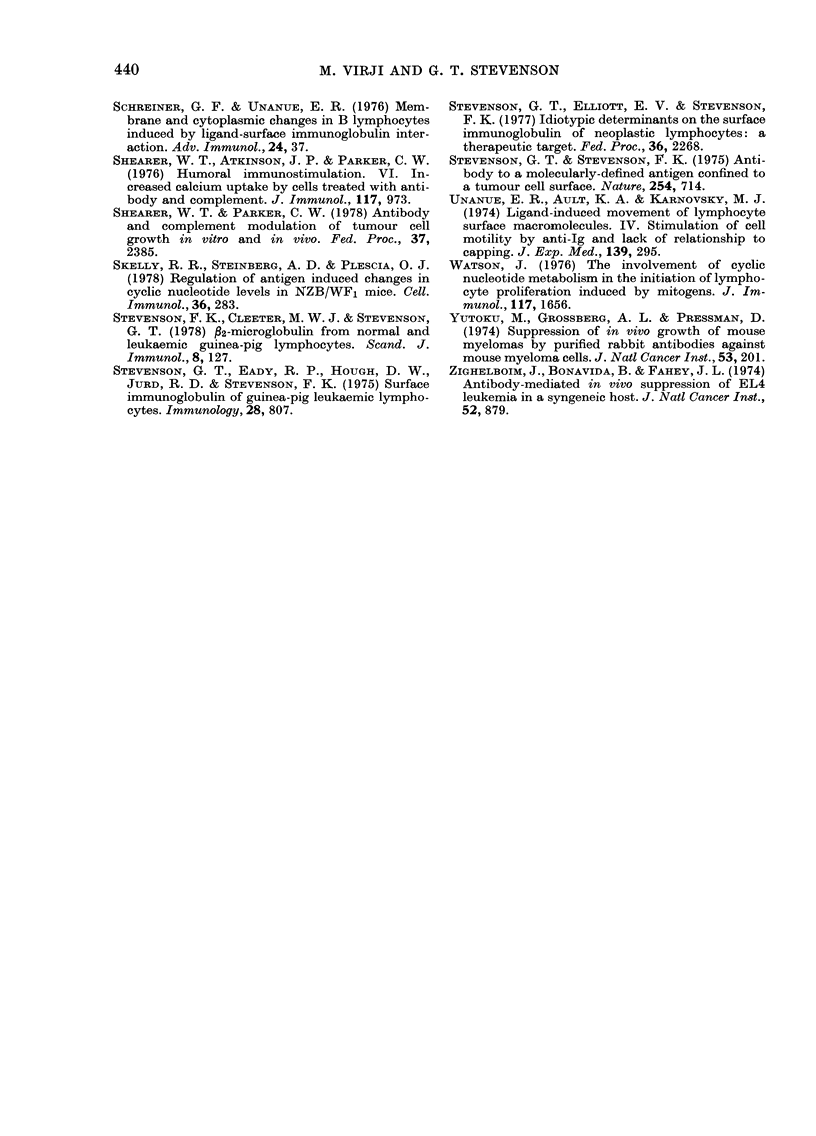# Antibody-induced changes in levels of cyclic adenosine monophosphate in leukaemic lymphocytes.

**DOI:** 10.1038/bjc.1979.77

**Published:** 1979-04

**Authors:** M. Virji, G. T. Stevenson

## Abstract

When L2C leukaemic B lymphocytes from guinea-pigs were incubated in vitro with antibody directed to their surface immunoglobulin (Ig), a rapid rise in intracellular adenosine 3':5'-phosphate (cyclic adenosine monophosphate, cAMP) was observed. Estimation of cAMP was by a protein-binding assay using bovine adrenal protein kinase. Increases up to 30-fold occurred within 30 seconds of incubation at 37 degrees C, to be succeeded by a fall which reached the basal level between 5 and 7 min. The response was proportional to the amount of antibody present. Cross-linking of surface Ig by the antibody was necessary, bivalent (Fab'gamma)2 from the antibody gave a rise in cAMP similar to that given by the parent molecule, whereas monomeric Fab'gamma was ineffective unless it was subsequently cross-linked by anti-antibody. The rise was too rapid to have required capping of the surface Ig for its induction. Not all perturbations of the plasma membrane by antibody induce such a surge in cAMP, since anti-beta2 microglobulin, also reacting with the lymphocyte surface, failed to alter cAMP concentration. The results emphasize that immunotherapy can be influenced by antibody altering the metabolic activity of target cells, quite apart from activation of immunological cytotoxic pathways.


*Br. J. Cancer* (1979) **39**, 434

### ANTIBODY-INDUCED CHANGES IN LEVELS OF CYCLIC ADENOSINE MONOPHOSPHATE IN LEUKAEMIC LYMPHOCYTES

M. VIRJI AND G. T. STEVENSON

*From the Tenovus Research Laboratory, General Hospital, Southampton*

Received 15 December 1978 Accepted 8 January 1979

**Summary.—When $L_2C$ leukaemic B lymphocytes from guinea-pigs were incubated *in vitro* with antibody directed to their surface immunoglobulin (Ig), a rapid rise in intracellular adenosine 3′:5′-phosphate (cyclic adenosine monophosphate, cAMP) was observed. Estimation of cAMP was by a protein-binding assay using bovine adrenal protein kinase. Increases up to 30-fold occurred within 30 seconds of incubation at 37°C, to be succeeded by a fall which reached the basal level between 5 and 7 min. The response was proportional to the amount of antibody present. Cross-linking of surface Ig by the antibody was necessary, bivalent $(Fab'\gamma)_2$ from the antibody gave a rise in cAMP similar to that given by the parent molecule, whereas monomeric $Fab'\gamma$ was ineffective unless it was subsequently cross-linked by anti-antibody. The rise was too rapid to have required capping of the surface Ig for its induction. Not all perturbations of the plasma membrane by antibody induce such a surge in cAMP, since anti-$\beta_2$ microglobulin, also reacting with the lymphocyte surface, failed to alter cAMP concentration. The results emphasize that immunotherapy can be influenced by antibody altering the metabolic activity of target cells, quite apart from activation of immunological cytotoxic pathways.**

FOLLOWING an initial study by Gorer (1942) there have been many reports that passively administered antibody can retard or ablate experimental lymphoid leukaemias and lymphomas (Motta, 1971; Hersey, 1973; Zighelboim *et al.*, 1974; O'Neill, 1976; Johnson *et al.*, 1977). The mechanisms involved are difficult to delineate, and present discussion (reviewed by Rosenberg & Terry, 1977) relies heavily upon simplified systems in which antibodies attack target cells *in vitro* with the recruitment of ancillary effectors: complement, K cells or macrophages. Much less attention has been paid to the effects of antibody alone, since this has long been known not to be frankly cytotoxic for nucleated target cells (Ross & Lepow, 1960). However, more subtle effects on normal and neoplastic lymphoid cells due to antibody alone have been recorded, such as altered motility and various metabolic changes (Unanue *et* al., 1974; Nishizawa *et al.*, 1977; Shearer & Parker, 1978). Furthermore, antibody has been reported to augment the susceptibility of neoplastic cells *in vitro* to X-rays and some cytotoxic drugs (Rubens *et al.*, 1975).

We have been studying leukaemias of B lymphocytes in man and guinea-pig, with particular regard to the tumour-specific antigens represented by idiotypic determinants on surface immunoglobulin (Ig) (Stevenson & Stevenson, 1975; Hough *et al.*, 1976). Passive anti-idiotype *in vivo* retards guinea-pig leukaemia (Stevenson *et al.*, 1977). For studies *in vitro* anti-Ig is used in the form of either anti-idiotype or the more readily available antibodies to constant determinants on the surface Ig.

Here we report that antibody alone, acting on the surface Ig of guinea-pig leukaemic lymphocytes, causes a rapid and dramatic rise in adenosine 3′: 5′-phosphate (adenosine cyclic monophosphate, cAMP). A signal is thereby transmitted to the cell which could initiate metabolic changes with important implications for immunotherapy.

### MATERIALS AND METHODS

*Cells.*—$L_2C$ lymphocytic leukaemia cells were obtained from a line provided in December 1972 by Dr E. Shevach of the National Institutes of Health, Bethesda, Md, U.S.A. A colony of Strain 2 guinea-pigs bred from animals supplied by the National Institutes of Health was used for passaging the cells *in vivo*. Blood from near-terminal animals was obtained by cardiac puncture and $L_2C$ cells were separated as described previously (Stevenson *et al.*, 1975). Such preparations contain $L_2C$ cells of $>95\%$ viability as judged by trypan-blue exclusion and $<2\%$ contaminating cells. The cells exhibit surface IgM, of light-chain class $\lambda$, with $\sim$50,000 monomeric IgM molecules per cell in the plasma membrane and no significant direct export of the Ig (Hough *et al.*, 1978).

*Immunoglobulins and antibodies.*—Antisera to guinea-pig Fab′$\gamma$ were raised in sheep as described by Stevenson *et al.* (1975). Such antisera are polyspecific for guinea-pig Ig classes by virtue of reactivity against light-chain determinants, and react with the surface IgM of $L_2C$ cells via its $\lambda$ chains. To obtain purified antibody, IgG from antiserum was passed through Sepharose 4B (Pharmacia) to which normal guinea-pig IgG had been coupled by the cyanogen bromide method of Axén *et al.* (1967). The antibody was eluted with 0·5M $NH_4OH$ and dialysed immediately against cold 0·2M Tris Buffer at pH 8. Some contaminating aggregates were removed by passage through an AcA 34 (L'industrie Biologique Francaise) column. The reactivity towards cell-surface immunoglobulin was determined by direct immunofluorescence and its specificity for Ig was demonstrated by blocking the reactivity with purified guinea-pig Ig.

Fab′$\gamma$ and (Fab′$\gamma)_2$ fragments were prepared from the above purified antibody by peptic digestion, respectively with or without subsequent reduction (Stevenson *et al.*, 1975). Residual IgG was removed from the (Fab′$\gamma)_2$ by passage through Sepharose 4B conjugated to rabbit anti-sheep Fc$\gamma$.

Purified antibody from sheep anti-guinea-pig $\beta_2$-microglobulin was a gift from F. K. Stevenson (Stevenson *et al.*, 1978). Its specificity was confirmed by the fact that its reaction with lymphocytic surfaces, assessed by direct immunofluorescence, was blocked by purified guinea-pig $\beta_2$-microglobulin (kindly supplied by I. Berggård, University of Lund, Sweden).

IgG from normal sheep sera was passed through Sepharose 4B conjugated to guinea-pig serum globulins to remove any natural antibody activity against guinea-pig Ig.

Antisera were raised in rabbits to normal sheep IgG by conventional means (Stevenson *et al.*, 1975). After preparation of rabbit IgG from the antisera, natural antibody activity to guinea-pig globulins was removed as described above.

*Cell cultures and extracts.*—Cell suspensions ($5\times10^7$/ml) were incubated with antibody at 37°C, normally in Eagle's minimal essential medium supplemented with 1% non-essential amino acids and 0·03% glutamine (supplemented MEM). The reaction was terminated by chilling rapidly in an ice bath. When intracellular cAMP levels were required, cultures were centrifuged at 4°C and the pellets only used. To determine total cAMP in a culture it was necessary to carry out the incubation in Dulbecco's medium to avoid materials interfering with the cAMP assay. In each case cell lysates were prepared by acidification (with HCl to 0·1M or perchloric acid to 1%) and heating at 80°C for 5 min.

*Estimation of cAMP.*—Lysates were neutralized with Tris or $KHCO_3$ and assayed by the protein-binding assay of Brown *et al.* (1971). The cAMP-binding protein, protein kinase from bovine adrenal cortex, was obtained from BDH Biochemicals (Poole, U.K.), and [$^3$H]-cAMP from the Radiochemical Centre (Amersham, U.K.). Other chemicals, including cAMP, were obtained from Sigma (London). All assays were in duplicate, giving ranges normally within 10% of the mean.

*Authentication of cAMP assay.*—Interference from other nucleotides was found to be minimal: ATP did not interfere when present in the assay at 100 $\mu$M, while 3′:5′-cCMP, 3′:5′-cIMP and 3′:5′-cGMP gave 50% inhibition of [$^3$H]-cAMP binding at 20 $\mu$M, 1·4 $\mu$M and 1·0 $\mu$M respectively. Identity of the nucleotide assayed was established by the phosphodiesterase treatment described by Watson (1976). Finally, assays on cAMP separated from cell extracts by sequential chromatography on Dowex 1 and thin-layer cellulose (Watson, 1976) agreed within 20% with assays on the crude extracts

### RESULTS

### *Basal levels of cAMP in $L_2C$ cells*

cAMP was estimated in cells freshly prepared and after incubation at $5 \times 10^7$ cells/ml in supplemented MEM at 37°C for up to 60 min. Values between 3 and 4 pmol/$10^7$ cells were obtained with no significant change occurring during incubation.

### *Antibody-induced changes in cAMP levels*

When $L_2C$ cells were incubated with antibody reacting with their surface Ig, a sharp, short-lived increase in the intracellular cAMP was seen. At 37°C the interaction resulted in a surge in the cAMP level which reached a peak within 30 seconds, followed by a rapid decline (Fig. 1). Incubations of cells from the same preparation at lower temperatures, exemplified in Fig. 1 by that at 25°C, showed quantitatively similar cAMP changes but over a longer period.

Experiments to demonstrate the dose-dependence of the antibody-induced rise in cAMP were carried out by adding varying amounts of antibody to the cells in Dulbecco's medium at 0°–2°C and then raising the temperature to 37°C. This device slowed the attainment of maximum cAMP concentrations and facilitated accurate measurements of the maxima. Total cAMP contents of the cultures were determined. As shown in Fig. 2, cAMP levels attained were a function of antibody concentration in the range studied.

When $L_2C$ cells were incubated in Dulbecco's medium as described above and only the culture fluid assayed, no significant amount of cAMP was detected. Furthermore, when total culture cAMP and intracellular cAMP were estimated in parallel experiments as described in Materials and Methods, identical values were obtained. This suggests that cAMP is degraded intracellularly after its in-

FIG. 1.—*In vitro* cAMP response in $L_2C$ cells on interaction with sheep anti-guinea-pig Fab′γ. $5 \times 10^7$ cells/ml in supplemented MEM were incubated with 1 mg/ml IgG ex sheep anti-guinea-pig Fab′γ at 37°C (■——■) or at 25°C (●— — —●). *Cells were equilibrated at the temperature of incubation prior to addition of the antibody.* ▲- - -▲: $5 \times 10^7$ cells/ml incubated at 25°C or 37°C without antibody. Each point represents the mean of assay duplicates, which usually agreed within 10%. The range shown for the incubations at 25°C comes from 2 experiments.

crease, with little if any being exported from the cells.

### *Requirement for multivalency of antibody*

$(Fab'\gamma)_2$ and Fab′γ fragments prepared from sheep anti-guinea-pig Fab′γ were tested in experiments similar to those described above for the whole antibody molecule. Whereas the response induced by $(Fab'\gamma)_2$ is comparable to that given by the whole antibody, Fab′γ failed to stimulate a significant response in $L_2C$ cells (Fig. 3). The requirement for cross-linking of the surface Ig was confirmed by a double-antibody technique (Fig. 4). When Fab′γ-coated $L_2C$ cells were incubated with rabbit anti-sheep IgG (1 mg/ml) at 37°C, there was a surge in cAMP levels similar to that observed previously with the bivalent antibody.

FIG. 2.—Dose-dependence of cAMP response in $L_2C$ cells. Average values of 3 estimations are plotted (s.e.$\not>$10% mean). $L_2C$ cells were cultured ($5 \times 10^7$ cells/ml) with antibody at different concentrations in Dulbecco's medium. After addition of antibody at 0°–2°C, the cultures were brought to 37°C for desired intervals, then chilled and HCl was added to a final molarity of 0·1. The cultures were heated at 80°C for 5 min and total cAMP was estimated. cultures contained sheep anti-guinea-pig Fab′γ at 500 μg/ml, ■——■; 125 μg/ml, ●——●; or 60 μg/ml, ▼——▼. Control samples contained normal sheep IgG (absorbed with guinea-pig globulin) at 500 μg/ml: ▲——▲.

FIG. 3.—Effect of antibody fragments on cAMP response in $L_2C$ cells. Antibody concentrations are in terms of antibody sites (2 per 150,000 mol. wt for IgG, 2 per 100,000 for (Fab′γ)$_2$, and 1 per 50,000 for Fab′γ). Cells were cultured at $5 \times 10^7$/ml in supplemented MEM with doubling dilutions of IgG ex purified sheep anti-guinea-pig Fab′γ (■——■), (Fab′γ)$_2$ fragment (●——●) or Fab′γ fragment (▼——▼) derived from the same antibody. After addition at 0°–2°C, cultures were brought to 37°C for 1·5 min. cAMP was extracted from the cell pellet in 0·1M HCl and assayed. Control incubations (▲- - -▲) contained normal sheep IgG absorbed with guinea-pig globulins. Average values of 2 estimations are plotted.

### *Effect of antibody to another surface constituent*

Purified sheep antibody to guinea-pig $\beta_2$-microglobulin, which was shown by immunofluorescence to react with the surface of $L_2C$ cells, gave no rise in the level of intracellular cAMP; neither upon incubation with the antibody alone at concentrations ranging from 2·5 to 400 μg/ml, nor in double-antibody incubations with rabbit anti-sheep IgG also present. Two types of double-antibody incubations were in fact carried out. In experiments with sequential addition, incubation occurred at 0°C with sheep anti-guinea-pig $\beta_2$-microglobulin (0·4 mg/ml), the antibody was removed by centrifugation, and finally rabbit anti-sheep IgG was added at 1 mg/ml and the temperature raised to 37°C for times ranging from 0·5 to 10 min. Alternatively, the cells were preincubated (0°C, 30 min) with 0·1 mg/ml or 0·4 mg/ml of the sheep antibody to guinea-pig $\beta_2$-microglobulin and the rabbit anti-sheep IgG (1 mg/ml) was added without the removal of the first antibody. In neither of these cases was there a significant change in cAMP levels. Parallel cultures, in which cells from the same preparation were exposed to anti-Ig (sheep anti-guinea-pig Fab′γ present at 0·5 mg/ml), gave the usual sharp increase in cAMP both in the presence and absence of the second antibody (present at 1 mg/ml).

FIG. 4.—Requirement for bivalency in the antibody-induced cAMP response of $L_2C$ cells. The cells were first incubated (0°C, 45 min) with Fab′γ fragment (0·3 mg/ml) derived from sheep IgG antibody to guinea-pig Fab′γ. The cells were then harvested by centrifugation (500 *g*, 4°C) and resuspended in cold supplemented MEM prior to incubation at 37°C in the absence (▲——▲) or presence (●——●) of 1 mg/ml of rabbit antibody to sheep IgG. The range shown for the incubations in the presence of the second antibody comes from 2 experiments. The rabbit antibody alone had no effect on uncoated cells nor on cells preincubated in the presence of normal sheep IgG.

*Effect of inhibitors of prostaglandin synthesis on the cAMP increase*

Neither acetyl salicylic acid nor indomethacin had any effect on the antibody-mediated rise in cAMP when present at up to 25 μg/ml in the incubation medium.

### DISCUSSION

The interaction of bivalent antibody with the surface Ig of $L_2C$ leukaemic lymphocytes has consistently provoked a dramatic surge in the level of intracellular cAMP. The requirement for antibody bivalence indicates that cross-linking of the surface Ig is required. The time needed to reach the cAMP maximum is similar to that required for patching, the earliest visible redistribution of surface Ig provoked by anti-Ig (Schreiner & Unanue, 1976). It seems to be too early for capping or appreciable endocytosis of the antibody-surface Ig complexes to have occurred. Both the rapidity of the reaction and its independence of inhibitors of prostaglandin synthesis distinguish it from a prostaglandin-dependent rise in cAMP described by Skelly *et al.* (1978) in normal spleen cells.

The dependence of the cAMP maximum on antibody concentration could reflect either the number of Ig receptors reacting or the tightness of cross-linking. It is noteworthy from our results with anti-$\beta_2$-microglobulin that a rise in cAMP is not an invariable sequel of bivalent antibody attaching to the cell surface, even if cross-linking be enhanced by anti-antibody forming a second layer. Ash *et al.* (1977) have reported that some membrane proteins, including Ig, form transmembrane linkages to actin and myosin upon being cross-linked. Whether this is necessary for the activation of adenyl cyclase, and consequent rise in cAMP, remains to be determined. Earp *et al.* (1977) have detected by fluorescence microscopy an association between surface Ig patches and submembranous cAMP in normal lymphocytes. It is difficult to relate this localized and prolonged accumulation of cAMP to the transient surge in total cellular cAMP found by us. Mechanisms responsible for the rapid subsidence of raised cAMP levels, among which activation of phosphodiesterase appears to be important, have been discussed by d'Armiento *et al.* (1972).

The second messenger concept (Robison *et al.*, 1971) states that a rise in cAMP leads to a variety of secondary metabolic effects. The significance of such effects in the present case has two facets: they might mimic physiological stimulation of normal B lymphocytes, and they might influence survival of neoplastic lymphocytes subjected to attack by antibody.

Schreiner & Unanue (1976) have reviewed the various metabolic changes which follow the action of anti-Ig on normal B lymphocytes. Such changes are of particular interest if they provide clues to the activation of B cells by antigen or their regulation by anti-idiotype. It is difficult, however, to extrapolate the present findings with leukaemic B lymphocytes to their normal analogues: the latter are usually not mitotically active, are difficult to isolate in pure culture, and appear highly dependent upon interactions with macrophages and T lymphocytes for their normal physiological responses.

The major significance we attach to our findings is that antibody can alter the behaviour of neoplastic cells in ways unrelated to recruitment of immunological killing mechanisms and varying with the precise molecular target on the cell surface. Antibody acting on neoplastic lymphocytes *in vitro* has previously been reported to yield a number of effects with evident implications for serotherapy *in vivo*: enhanced calcium uptake (Shearer *et al.*, 1976), inhibition of migration (Cochran *et al.*, 1973), and enhanced nucleoside uptake and mitosis (Shearer & Parker, 1978). Anti-Ig itself has been shown to inhibit migration *in vitro* by guinea-pig and human leukaemic lymphocytes (Stevenson & Stevenson, 1975; Hough *et al.*, 1976). It will be of interest to determine whether any of these longer-term effects are mediated via cyclic nucleotide mechanisms. Such metabolic data could also be relevant to the synergism reported to exist sometimes between antibody and drugs (Rubens *et al.*, 1975). Finally, direct metabolic effects of antibody could have a role in the well known phenomenon of tumour enhancement, the stimulation of growth *in vivo* by antibody (*e.g.* Yutoku *et al.*, 1974): this is often attributed to antibody blocking a cell-mediated attack on the tumour cells, but a direct stimulatory effect by the antibody might sometimes be more important.

This work has been supported by Tenovus of Cardiff, the Cancer Research Campaign and the Wessex Regional Health Authority. We are grateful for the technical assistance of Miss Alison Tutt, Mrs Maureen Quinton and Mrs Maureen Power.

### REFERENCES

Ash, J. F., Louvard, D. & Singer, S. J. (1977) Antibody-induced linkages of plasma membrane proteins to intracellular actinomyosin-containing filaments in cultured fibroblasts. *Proc. Natl Acad. Sci., USA*, **74**, 5584.

Axén, R., Porath, J. & Ernback, S. (1967) Chemical coupling of peptides and proteins to polysaccharides by means of cyanogen halides. *Nature*, **214**, 1302.

Brown, B. L., Albano, J. D. M., Ekins, R. P., Sgherzi, A. M. & Tampion, W. (1971) A simple and sensitive saturation assay method for the measurement of adenosine 3′:5′-cyclic monophosphate. *Biochem. J.*, **121**, 561.

Cochran, A. J., Kiessling, R., Klein, E., Gunven, P. & Foulis, A. K. (1973) Human tumour cell migration. *J. Natl Cancer Inst.*, **51**, 1109.

D'Armiento, M., Johnson, G. S. & Pastan, I. (1972) Regulation of adenosine 3′:5′-cyclic monophosphate phosphodiesterase activity in fibroblasts by intracellular concentrations of cyclic adenosine monophosphate. *Proc. Natl Acad. Sci., USA*, **69**, 459.

Earp, H. S., Utsinger, P. G., Yount, W. J., Logue, M. & Steiner, A. L. (1977) Lymphocyte surface modulation and cyclic nucleotides. I. Topographic correlation of cyclic adenosine 3′:5′-monophosphate and immunoglobulin immunofluorescence during lymphocyte capping. *J. Exp. Med.*, **145**, 1087.

Gorer, P. A. (1942) The role of antibodies in immunity to transplanted leukaemia in mice. *J. Pathol. Bacteriol.*, **54**, 51.

Hersey, P. (1973) New look at antiserum therapy of leukaemia. *Nature (New Biol.)*, **244**, 22.

Hough, D. W., Chapple, J. C., Stevenson, F. K. & Stevenson, G. T. (1978) Further studies of immunoglobulin synthesis by guinea-pig leukaemic lymphocytes. *Immunology*, **34**, 889.

Hough, D. W., Eady, R. P., Hamblin, T. J., Stevenson, F. K. & Stevenson, G. T. (1976) Anti-idiotype sera raised against surface immunoglobulin of human neoplastic lymphocytes. *J. Exp. Med.*, **144**, 960.

Johnson, R. J., Pasternack, G. R. & Shin, H. S. (1977) Antibody-mediated suppression of tumor growth. I. Suppression by murine IgG1 isolated from alloantiserum. *J. Immunol.*, **118**, 489.

Motta, R. (1971) Passive immunotherapy of leukaemia and other cancer. *Adv. Cancer Res.*, **14**, 161.

Nishizawa, Y., Kishimoto, T., Kikutani, H. & Yamamura, Y. (1977) Induction and properties of cytoplasmic factor(s) which enhance nuclear non-histone protein phosphorylation in lymphocytes stimulated by anti-Ig. *J. Exp. Med.*, **146**, 653.

O'Neill, G. J. (1976) Control of an EL4 lymphoma in nude mice by passively administered antibody. *Eur. J. Cancer*, **12**, 749.

Robison, G. A., Butcher, R. W. & Sutherland, E. W. (1971) Cyclic AMP and hormone action. In *Cyclic AMP*. London and New York: Academic Press. p. 22.

Rosenberg, S. A. & Terry, W. D. (1977) Passive immunotherapy of cancer in animals and man. *Adv. Cancer Res.*, **25**, 323.

Ross, A. & Lepow, I. H. (1960) Studies on immune cellular injury. I. Cytotoxic effects of antibody and complement. *J. Exp. Med.*, **112**, 1085.

Rubens, R. D., Vaughan-Smith, S. & Dulbecco, R. (1975) Augmentation of cytotoxic drug action and X-irradiation by antibodies. *Br. J. Cancer*, **32**, 352.

SCHREINER, G. F. & UNANUE, E. R. (1976) Membrane and cytoplasmic changes in B lymphocytes induced by ligand-surface immunoglobulin interaction. *Adv. Immunol.*, **24**, 37.

SHEARER, W. T., ATKINSON, J. P. & PARKER, C. W. (1976) Humoral immunostimulation. VI. Increased calcium uptake by cells treated with antibody and complement. *J. Immunol.*, **117**, 973.

SHEARER, W. T. & PARKER, C. W. (1978) Antibody and complement modulation of tumour cell growth *in vitro* and *in vivo*. *Fed. Proc.*, **37**, 2385.

SKELLY, R. R., STEINBERG, A. D. & PLESCIA, O. J. (1978) Regulation of antigen induced changes in cyclic nucleotide levels in NZB/$WF_1$ mice. *Cell. Immunol.*, **36**, 283.

STEVENSON, F. K., CLEETER, M. W. J. & STEVENSON, G. T. (1978) $\beta_2$-microglobulin from normal and leukaemic guinea-pig lymphocytes. *Scand. J. Immunol.*, **8**, 127.

STEVENSON, G. T., EADY, R. P., HOUGH, D. W., JURD, R. D. & STEVENSON, F. K. (1975) Surface immunoglobulin of guinea-pig leukaemic lymphocytes. *Immunology*, **28**, 807.

STEVENSON, G. T., ELLIOTT, E. V. & STEVENSON, F. K. (1977) Idiotypic determinants on the surface immunoglobulin of neoplastic lymphocytes: a therapeutic target. *Fed. Proc.*, **36**, 2268.

STEVENSON, G. T. & STEVENSON, F. K. (1975) Antibody to a molecularly-defined antigen confined to a tumour cell surface. *Nature*, **254**, 714.

UNANUE, E. R., AULT, K. A. & KARNOVSKY, M. J. (1974) Ligand-induced movement of lymphocyte surface macromolecules. IV. Stimulation of cell motility by anti-Ig and lack of relationship to capping. *J. Exp. Med.*, **139**, 295.

WATSON, J. (1976) The involvement of cyclic nucleotide metabolism in the initiation of lymphocyte proliferation induced by mitogens. *J. Immunol.*, **117**, 1656.

YUTOKU, M., GROSSBERG, A. L. & PRESSMAN, D. (1974) Suppression of *in vivo* growth of mouse myelomas by purified rabbit antibodies against mouse myeloma cells. *J. Natl Cancer Inst.*, **53**, 201.

ZIGHELBOIM, J., BONAVIDA, B. & FAHEY, J. L. (1974) Antibody-mediated *in vivo* suppression of EL4 leukemia in a syngeneic host. *J. Natl Cancer Inst.*, **52**, 879.